# Camel milk peptide improves wound healing in diabetic rats by orchestrating the redox status and immune response

**DOI:** 10.1186/s12944-015-0136-9

**Published:** 2015-10-24

**Authors:** Hossam Ebaid, Bahaa Abdel-salam, Iftekhar Hassan, Jameel Al-Tamimi, Ali Metwalli, Ibrahim Alhazza

**Affiliations:** Department of Zoology, College of Science, King Saud University, P.O. Box 2455, Riyadh, 11451 Saudi Arabia; Department of Zoology, Faculty of Science, El-Minia University, El-Minia, Egypt; Department of Biology, College of Science and Humanities in Quwiaya, Riyadh, 11961 Saudi Arabia; Department of Food Science, College of Agriculture and Food Science, King Saud University, Riyadh, Saudi Arabia; Department of Dairy, Faculty of Agriculture, El-Minia University, El-Minia, Egypt

**Keywords:** Diabetic model, Camel milk peptide, Oxidative stress, TNF-α, IL-1β, Wound healing

## Abstract

**Background:**

Diabetes mellitus alters oxidative stability and immune response. Here, we investigated the impact of a peptide extracted from camel milk (CMP) on the oxidative status, transcription factor kappa-B (NF-kB) and inflammatory cytokine in diabetic wounds.

**Methods:**

Rats were assigned into three groups: control, diabetic induced (DM) and diabetic induced with multiple doses of CMP for a week (DM-CMP).

**Results:**

DM showed a sharp decline in the activity of major antioxidant enzymes such as superoxide dismutase (SOD), catalase (CAT) and glutathione (GSH) compared to the control. The DM-CMP group, however, showed a noticeable replenishment in the activity of these enzymes compared to the DM group. The CMP-treated group also showed a normal level of lipid peroxidation marker (MDA) compared to the DM rats. Furthermore, ELISA analysis of serum TNF-α protein showed an elevated level in diabetic rats in comparison to control serum. However, RT-PCR analysis of locally wounded skin tissues revealed that diabetes down-regulates the RNA expression of both TNF-α and MIF genes in comparison to the control samples but that CMP was found to restore RNA expression significantly. Although it was elevated in CMP-treated rats after one day of wound incision, the NF-kB protein level was significantly decreased seven days after the incision in comparison to the animals in the diabetic group.

**Conclusion:**

CMP, therefore, can be seen an effective antioxidant and immune stimulant that induces oxidative stability and speeds up wound healing in diabetic model animals, making it a potential adjuvant in improving wound healing in those with diabetic conditions.

## Background

Oxygen free radicals are normally balanced by the presence of adequate endogenous antioxidant defences [1]. An imbalance between endogenous/exogenous oxidants and the antioxidant system in a living organism is called oxidative stress. This type of stress has been implicated in the pathogenesis of various diseases including diabetes mellitus [2–4]. One of the major concerns in respect to diabetic patients is their prolonged inflammatory period that delays, or exerts a derogatory effect, on the natural wound healing process.

Normally, wound healing is initiated by an inflammatory phase that is followed by a proliferation of fibroblasts and endothelial cells, and then by the production and reorganization of the extracellular matrix, leading to repair or regeneration. The inflammatory phase provokes the recruitment of leukocytes that produce growth factors and remove debris from the wound [5–7]. The healing process requires an interaction between inflammatory cells and biochemical mediators, which is stimulated by a number of mitogens and chemotactic factors [8].

IL-1β and TNF-αare critical to the normal inflammatory phase of wound healing, while NF-kB is required for the induction of pro-inflammatory cytokines, such as IL-1β, TNF-α and IL-6 [9]. IL-1β and TNF-α modulate the expression of the chemokines and adhesion molecules necessary for the recruitment of inflammatory cells to the site of injury [10, 11]. The inflammatory phase recruits leukocytes that produce growth factors and remove debris from the wound [5]. Impairment of leukocyte recruitment is associated with delayed wound healing [7]. Neutrophils release highly active antimicrobial substances, proteinases [12] and inflammatory cytokines which also have crucial roles in the healing of wounds.

Our previous work based on the proteins derived from camel milk have demonstrated their enhanced wound healing capacity in diabetic as well as older animals [13–15]. Additionally, our recent study [16] revealed the ability of this protein to direct immune processes and its ability to trigger the proliferation of peripheral blood mononuclear cells (PBMCs) [17]. In the present piece of work, we hypothesize that wound healing in diabetic animals could be improved by supplementing camel milk derived protein fractions. Thus, the camel milk proteins were enzymatically digested into different peptide fractions in order to find the active one to improve the diabetic wound healing.

## Materials and methods

### Enzymatic and degree of hydrolysis of camel milk whey proteins

Camel milk was obtained from a camel breed (Majaheem) from the Najd region in Saudi Arabia. Camel milk was used to isolate the whey proteins as previously described [13–15]. Briefly, Skim milk was acidified to pH 4.3 using 1 M HCl. The precipitated casein was removed by centrifugation, and the supernatant containing the whey protein was saturated with ammonium sulfate (70 % saturation) and incubated overnight at 4 °C. The precipitated whey protein was collected by centrifugation and dialyzed against distilled water for 48 h at 4 °C using a Spectra/Pro® Membrane, MWCO 6000–8000 Da. The obtained dialyzate was lyophilized using a Unitop 600SL, [Virtis Company, Gardiner, New York 12525 USA] and were kept at −20 °C until use. The dialyzate containing non-denatured whey protein was freeze-dried and refrigerated until use.

A 2.5 % whey proteins solution was suspended in water at pH 7.0 using 1 mol NaOH. After complete solublization, the temperature was adjusted at 37 °C and the trypsin enzyme was added to proteins in ratio 1/100 and incubated. The enzyme inactivation was achieved by heating the samples in boiling water bath for 5 min. Samples were cooled under running tap water followed by their storage in the refrigerator until usage. Degree of protein hydrolysis was determined using the ortho-phthaldialdehyde based method. Then protein fractions were independently tested to their bioactivities (wound closure rate, histological features of cutaneous epidermal and dermal events, and oxidative stability). Among them, the peptide fraction 1 (camel milk peptide: CMP) was chosen for wound healing experiments in vivo.

### Ethical approval and preparation of un-denatured camel milk whey proteins

Camel milk was obtained from a camel breed (Majaheem) from the Najd region (Alazeria farm; GPS: 300 02 47/ 300 02 27) in Saudi Arabia. Specific permissions were not required for activities in this private farm as this study did not involve endangered or protected species. Regarding experimental animals, all procedures were conducted in accordance with the standards set forth under the guidelines for the care and use of experimental animals by the Committee for the Purpose of Control and Supervision of Experiments on Animals and the National Institutes of Health. The study protocol (care and handling of experimental animals) was approved by the Animal Ethics Committee of the Zoology Department in the College of Science at King Saud University, Riyadh (KSA).

### Diabetes induction and wound incision

Diabetes was induced by a single injection of freshly dissolved streptozocin (STZ) at the standardised dose of 60 mg/kg of body weight in a 0.1 Mcitrate buffer (pH 4.5) into the peritoneum region of the rats. Control rats received only equal volume of citrate buffer. After seven days, the rats were screened for serum glucose levels in which a serum glucose level ≥ 200 mg/dl after 2 h of glucose intake were considered as diabetic and selected for further studies.

Rats were anaesthetized with isoflurane. Their backs were gently shaved with sterilization using an alcohol swab. The wound biopsy model used in this experiment has been described previously [18] with little modification. The shaved skin was pinched and folded, and the wound was punched through the full thickness of the folded skin to form a 2 × 5 mm rectangle below the shoulder blades in each rat.

### Experimental design

Twenty four healthy male Sprague–Dawley rats, 8 weeks old and weighting 150 g to 200 g, were purchased from the Animal Breeding Center in college of pharmacy, King Saud University (Riyadh, KSA). This murine model was used to mimic the pathophysiological features seen in human diabetic wounds as previously described [19]. The animals were assigned to three groups: 1) the first group was remained as a wounded non-diabetic control group (CN) and was given phosphate buffered saline, 2) the second group was a wounded diabetic group (DM) that was treated with phosphate buffered saline, 3) the third group was a wounded diabetic group that was orally supplemented with camel milk peptide (CMP) at a dose of 25 mg/kg of body weight for 7 days (was daily administered as a single dose by gavage for 7 days) during wounding healing. Additional supplementary groups such as diabetic rats treated with whey protein fraction 2 and the non-injuried diabetic groups were studied for confirming the results of the three main groups. However, data from supplementary groups are not included in this study.

Seven animals from each group were sacrificed under mild diethyl ether anesthesia after 1st and 7th day after the wounding.

### Evaluation of the intensity of histochemical staining

All expression patterns were analyzed by two independent investigators with histology expertise on skin and pancreas. The expression ranged from 1 = low (<10 %), 2 = medium (10–25 %), 3 = high (26–70 %), 4 = very high (71 %) were followed under the present study [20].

### Measurement of wound closure

The procedure for measuring wound closure was conducted according to previously described by Lim et al. [9]. Wounds from individual rats were digitally photographed every day. A standard rectangle equivalent in size to the initial wound area was drawn beside the wound and used as a reference. Wound size was calculated by determining the area of the wound each day in comparison to the area of the standard rectangle. Wound closure was expressed as the ratio of the initial wound size to the wound area (each day after wounding). A higher ratio indicates faster wound closure.

### Collection of blood and tissue samples

Two blood samples were immediately collected. The first sample was used for serum analysis. Plasma was isolated from the second sample using EDTA (ethylenediaminetetra acetic acid) as an anticoagulant. Samples of plasma and serum were separated for analysis by centrifuging the blood for 15 min at 2000 rpm.

### Assay of Antioxidant Enzymes (SOD and CAT)

The activity of different antioxidant enzymes in the liver homogenates was assayed with standard protocols. Cu Zn superoxide dismutase (CuZnSOD) was assayed by autoxidation of pyrogallol [21] while catalase (CAT) was assayed by decomposition of hydrogen peroxide [22].

### Estimation of GSH and MDA levels

The level of reduced glutathione (GSH) was estimated in the liver homogenates by method of Jollow et al. [23]. The extent of lipid peroxidation was estimated by the method of Buege and Aust [24] involving the measurement of total malondialdehyde (MDA).

### ELISA assay for the inflammatory cytokines TNF-α and NF-kB

Sera and liver homogenates were tested for TNF-α and NF-kB, respectively by ELISA according to the manufacturer’s instructions for the corresponding rat immunoassay kits (Abcam, USA). The optical densities were measured at 405 nm. The detection limits were set according to the log-log correlative coefficient of the standard curve.

### RT-PCR analysis of RNA expression of MIF and TNF- α in wounded skin

Quantification of mRNA expression by real-time polymerase chain reaction cDNA from the above preparation was subjected to PCR amplification using 96-well optical reaction plates in the ABI Prism 7500 System (Applied Biosystems®). The 25-μl reaction mixture contained 0.1 μl of 10 μM forward primer and 0.1 μl of 10 μM reverse primer (40 μM final concentration of each primer), 12.5 μl of SYBR Green Universal astermix, 11.05 μl of nucleasefree water, and 1.25 μl of cDNA sample. The primers used in the current study were chosen from pubmed com. The RT-PCR data was analyzed using the relative gene expression method, as described in Applied Biosystems ® User Bulletin No. 2. The data are presented as the fold change in gene expression normalized to the endogenous reference gene and relative to a calibrator.

### Statistical analysis

The statistical analysis was performed using MINITAB, State College, PA, Version 13.1, 2002. The data were normally distributed with homogeneous variances. One-way ANOVA statistical measure was used to determine the overall effect of each treatment. This measure was supplemented by individual comparison between the different treatments using Tukey’s method for pairwise comparisons. The results were expressed as mean (M) ± standard deviation (SD). Only statistically significant differences with *P* < 0.05 were found between the treatment group and the control, and between the treatment group and the diabetic group considered.

## Results

### Electrophoresis pattern of the hydrolysed whey proteins

**Fig. 1:**
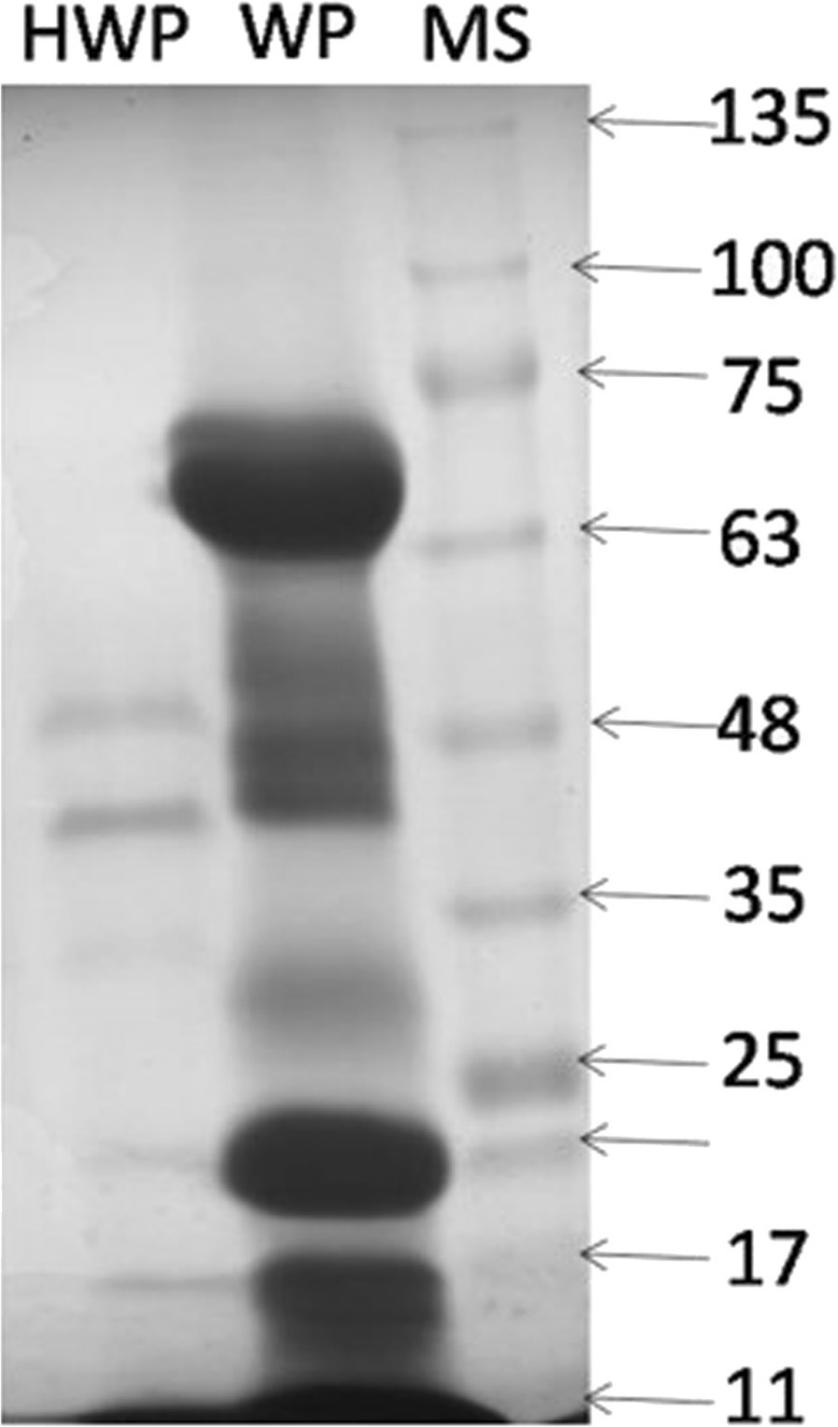
Electrophoresis pattern of native and hydrolysed whey proteins: MS: marker with molecular weight ranged from 135 to 11 KDa; WP: native whey proteins; HWP: hydrolysed whey proteins with trypsin enzyme for 3 h at 37

Electrophoresis-pattern clearly revealed that the camel whey proteins extensively hydrolysed during incubation time for 3 h with trypsin enzyme (Fig. 1). After hydrolysis whey protein hydrolysates were separated by centrifugation into several fractions; the total unhydrolysed whey protein, the hyrolysed whey protein, long chain peptide and the camel milk peptide1 (CMP). Different fractions were independently tested. Among them, the CMP (3 kDa) was chosen for wound healing experiments in vivo.

### CMP enhanced wound closure rate in diabetic models

**Fig. 2:**
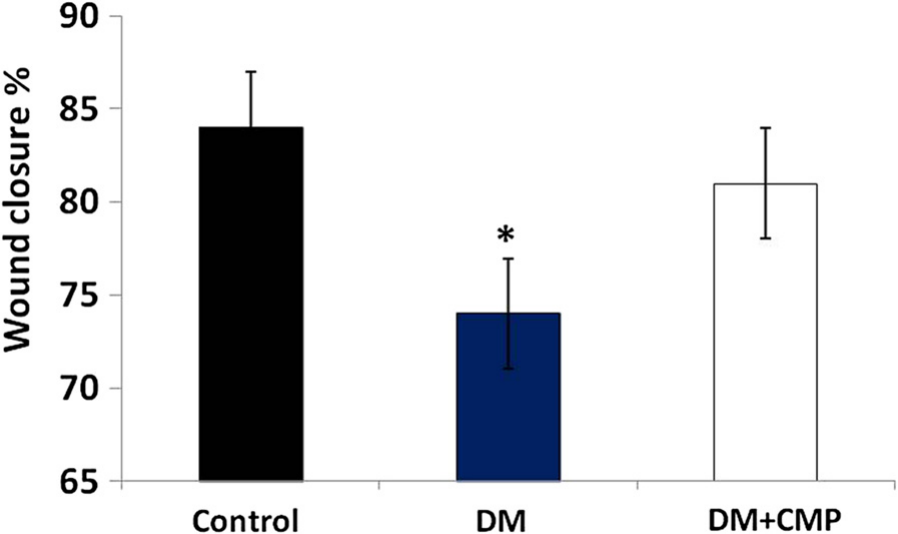
Wound closure rate in non-diabetic normal rats, diabetic rats (DM) and diabetic rats supplemented with CMP. All the values have been expressed as mean ± SEM for six different preparations. * indicates significantly different from the control group. # indicates significantly different from the diabetic group (1D)

Morphologically, no significant differences were observed between the different rat groups on day 1. As the experiment progressed it was evident that the time required to heal wounds was significantly shortened in the CMP treated rats comparing to the untreated diabetic rats. All the wounded diabetic animals fed CMP achieved complete healing by day 7 (Fig. 2).

### Effect of CMP on collagen deposition in the dermis of the wounded tissues

**Fig. 3:**
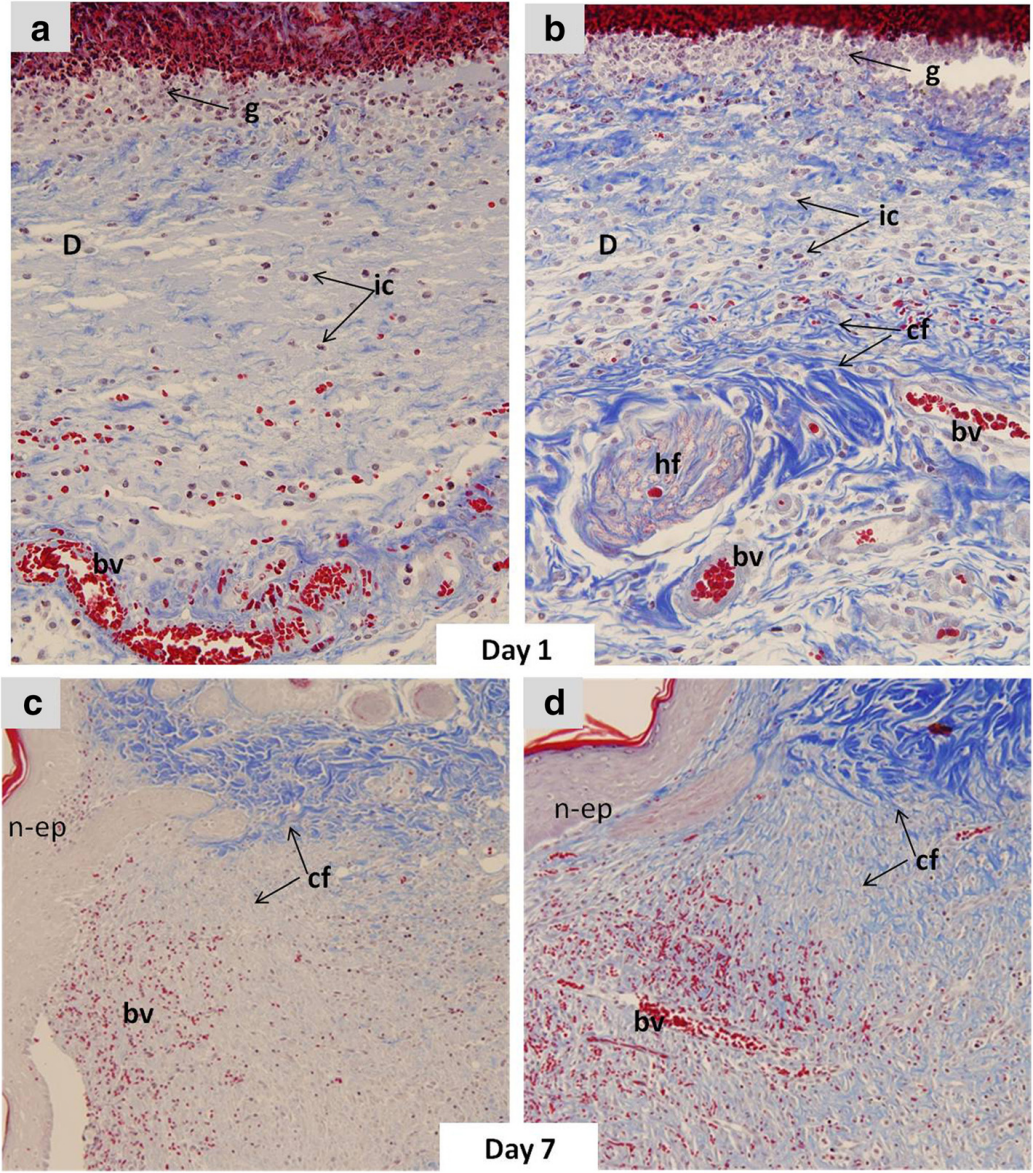
Representative photographs from the vertical sections of the wound sites from diabetic (**a**) and diabetic-treated with CMP (**b**) rats one day after incision (MTS × 400), and diabetic (**c**) and diabetic-treated with CMP (**d**) seven days after incision (MTS × 200), showing dermis (*d*), granulation (*g*), inflammatory cells (*ic*), blood vessels (*bv*) and new epidermis (*n-ep*)

Histological examination demonstrated that wounded tissues from the diabetic rats appeared disturbed one day after wounding (Fig. 3), while those of the CMP diabetic rats seemed similar to the normal tissues. One of the most indicative elements of dermal recovery is the rate of collagen fibril deposition and Mallory Trichrome stain demonstrated that in the CMP-treated rats there were a moderate number of collagen fibrils and the collagen bundles were organized in a more regular fashion than in the untreated diabetic rats, which tended to be asymmetrically distributed. Dermal regeneration in rats supplemented with CMP was characterized by fibroblasts and well-developed symmetrically distributed collagen bundles (Fig. 3). Table 1 summarizes the histological changes detected in dermal regions from different wounded groups.

**Table 1:** The expression of different parameters in the dermis of the wounded region, one and seven days after wound incision

Groups	Collagen fibres		Hair follicles		Dermal depth		New blood vessels		Number of Neutrophil		Inflammatory cells	
	D1	D7	D1	D7	D1	D7	D1	D7	D1	D7	D1	D7
Control	2	3	1	3	1	3	1	3	4	1	4	1
DM	1	2	1	2	1	2	1	2	2	3	2	3
DM-CMSP	2	3	1	3	1	4	1	4	4	1	4	1

1 = low (<10 %), 2 = medium (10–25 %), 3 = high (26–70 %), 4 = very high (71 %)

### The effect of camel milk peptide on oxidative stress

**Fig. 4:**
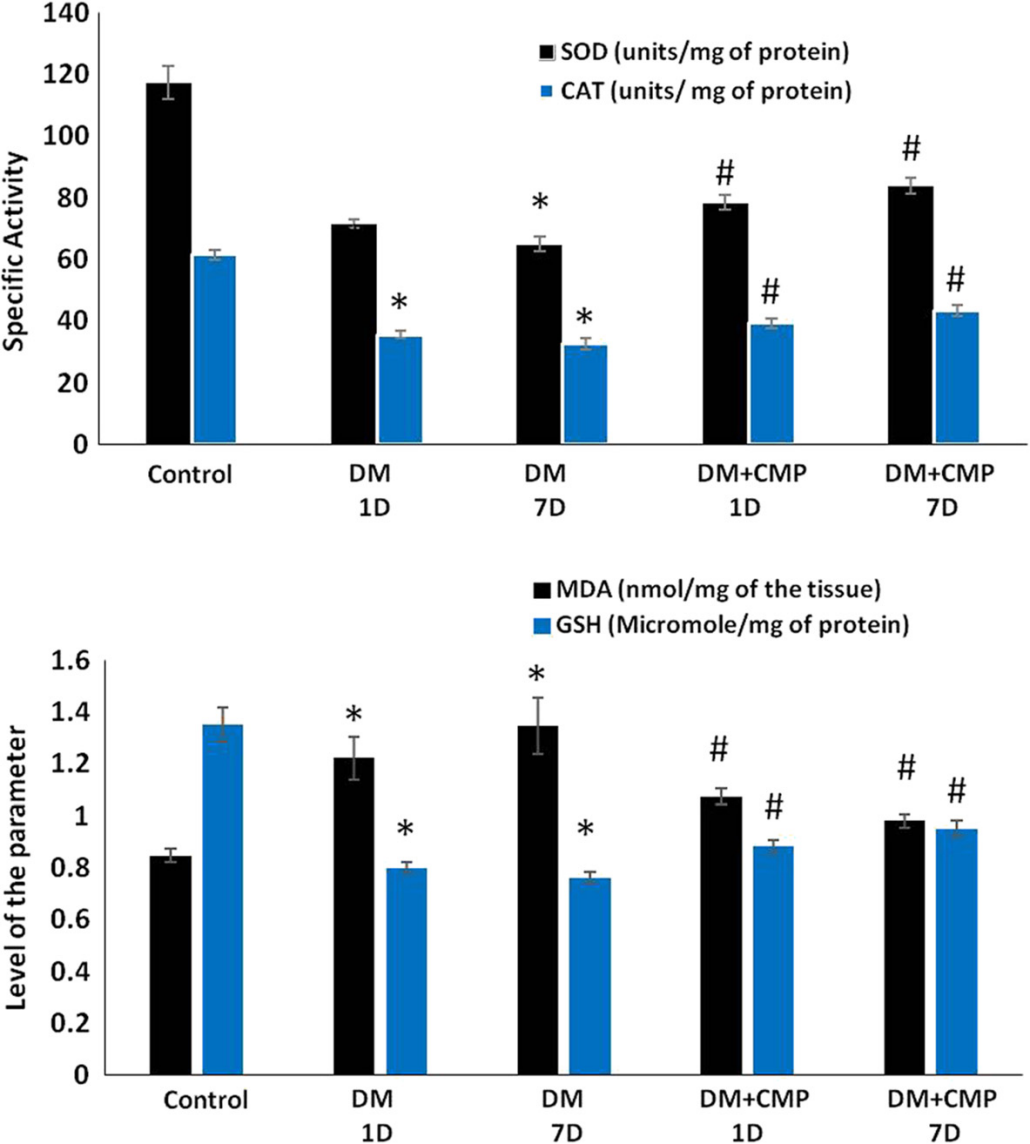
Specific activity of the enzymes-SOD and CAT, and level of MDA and reduced GSH (micromoles per mg of the protein in the samples). All the values have been expressed as mean ± SEM for six different preparations. * indicates significantly different from the control group. # indicates significantly different from the diabetic group (1D)

In the present study, the assessment of oxidative stress was based on measuring the specific activity of key antioxidant enzymes, SOD and CAT, along with estimation of the level of reduced GSH and MDA in the liver samples of different treatment groups (Fig. 4). All the diabetic induced groups (DM-1D and DM-7D) were compared to the control normal group while all CMP-treated diabetic groups (DM + CMP-1D and DM + CMP-7D) were compared to the DM-1D group.

SOD is considered to be the most important antioxidant enzyme in the assessment of redox status in living organisms. Group DM-1D showed a sharp decline in its specific activity (by 39.04 %) and decreased further as the experiment progressed so that it was 44.54 % lower than the control by seven days after wounding (DM-7D). Group DM + CMP-1D, however, demonstrated an increase in its activity by 9.8 % while DM + CMP-7D showed a significant increase in activity by 17.54 %, compared to DM-1D (Fig. 4).

CAT is also a very prominent antioxidant enzyme. Its activity was found to be decreased by 42.19 % in DM-1D and by 47.15 % in the case of DM-7D, compared to control rats. Among the peptide treated groups, DM + CMP-1Dshowed an increase in its activity of 10.27 %, whereas DM + CMP-7D demonstrated a prominent increase of 22 % compared to DM-1D (Fig. 4).

In tandem with the enzymes mentioned above, GSH is one of the most important cellular reductants and plays a vital role in maintaining the normal redox status in a cell. DM-1D showed a decrease in GSH of 40.87 %, while DM-7D exhibited a decline of 43.68 %, compared to the control. DM + CMP-1D, meanwhile, demonstrated an increase in GSH level of 10.25 %, which further risen to 19 % in DM + CMP-7D, compared to DM-1D (Fig. 4).

MDA is one the most stable oxidative products post lipid peroxidation in vivo as well as in situ, and is considered a very reliable parameter to assess the oxidative burden in biological systems. In the present study, DM-1D showed a marked increase in MDA level, by 44.10 %, while DM-7D displayed an increase of 58.84 % compared to the control. Among the peptide treated groups, DM + CMP-1D showed a decline in its level by 12.02 %, while DM + CMP-7D demonstrated a 19.72 % decrease compared to DM-1D (Fig. 4).

### The effect of the camel milk peptide on TNF-α and NF-kB protein levels

**Fig. 5:**
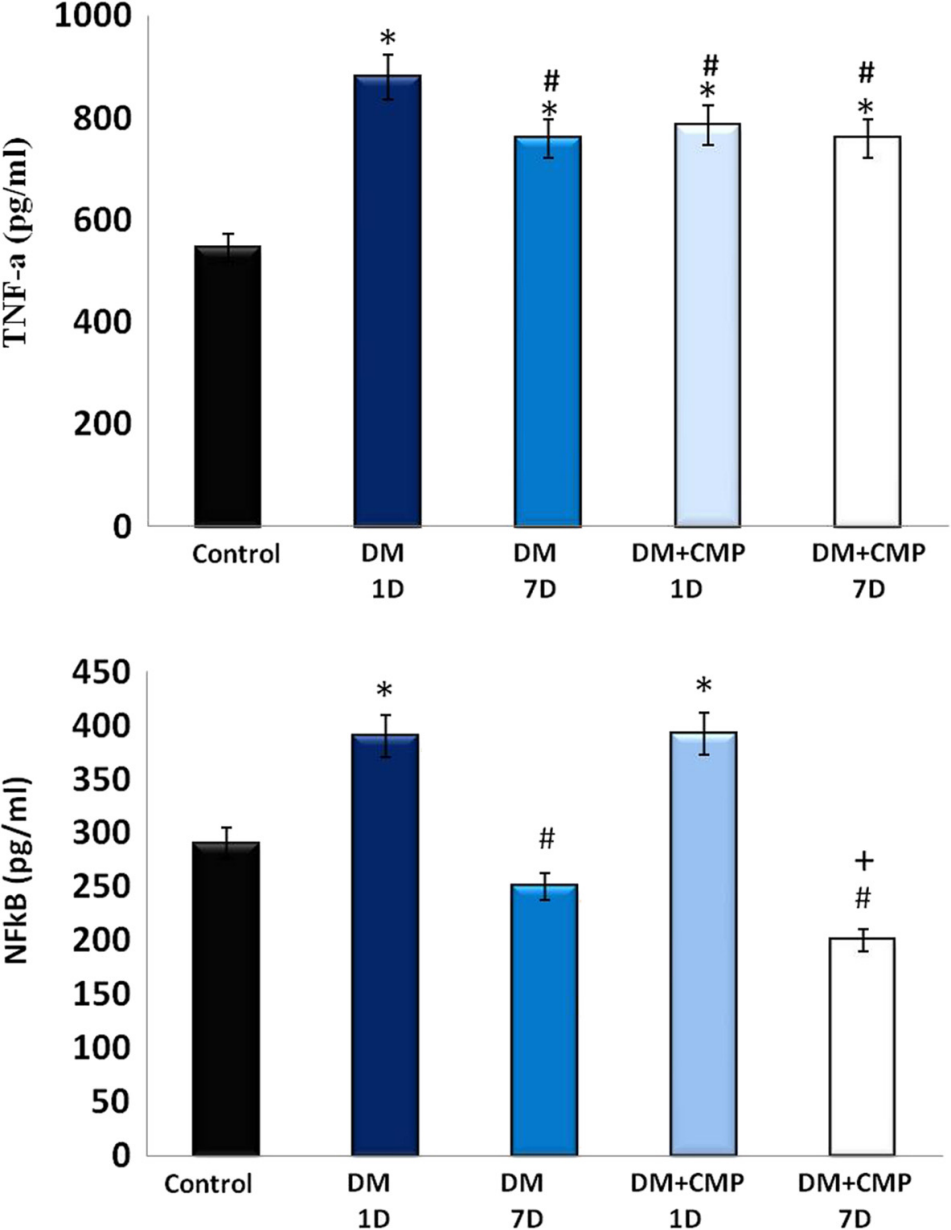
ELISA estimation of both IL-1β and TNF-α levels at days 1, 7 in wounded normal rats, wounded diabetic rats (DM) and wounded diabetic rats supplemented with CMP. All the values have been expressed as mean ± SEM for six different preparations. * indicates significantly different from the control group. # indicates significantly different from the diabetic group (1D). + indicates significantly different from the diabetic group (7D)

It was necessary to investigate the level of critical inflammatory cytokines like TNF-α and its initial molecule, NF-kB in normal wound healing, since these are considered to be strongly related to oxidative status. ELISA analysis of serum TNF-α protein level showed that it was significantly elevated in diabetic induced groups (DM-1D and DM-7D) in comparison to the control in their serum samples. While the TNF-α protein level remained considerably higher in CMP- diabetic rats as well as in the untreated diabetic group DM-1D and DM-7D compared to the control. However, DM + CMP-1D and DM + CMP-7D groups showed noticeable lower level of TNF- α with respect to the DM-1D group (Fig. 5).

Besides, we estimated the level of NF-kB, which is the initial molecule of TNF-α, in the liver samples. CMP was found to elevate its level significantly after one day of the incision (inflammatory phase) as shown by the group- DM + CMP-1D in comparison to the control group. Interestingly, the peptide of interest, CMP was found to restore the level of the protein to the normal level after seven days of the incision as evidenced in the group- DM + CMP-7D (Fig. 5).

### The effect of CMP on MIF and TNF-α RNA expression

**Fig. 6:**
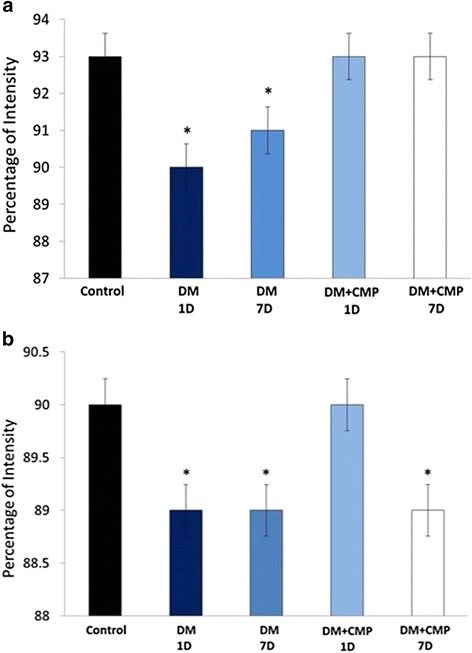
A: Histogram of real time PCR gene expression of both MIF (**a**) and TNF-α (**b**) levels at days 1, 7 in wounded normal rats, wounded diabetic rats (DM) and wounded diabetic rats supplemented with CMP. * indicates significantly different from the control group. # indicates significantly different from the diabetic group (1D)

After estimating TNF-α and its initial molecule, NF-kB in the serum and tissues respectively, we were interested to quantify the RNA expression of TNF-α and MIF in the vicinity of wounded regions in the three groups- one and seven days after incision. TNF-α and MIF are recognized as critical molecules in arresting infections by modulation of inflammatory responses. Thus, in the present study, assessment of TNF-α and MIF was needed to evaluate the inflammatory phase. Results of RT-PCR revealed that there was down-regulation of the RNA expression of TNF-α genes in diabetic rats after one and seven days of incision of the wound in comparison to the control samples while MIF was found upregulated in CMP-treated groups as compared to the DM-1D with a prominent tendency towards the control levels. Hence, CMP was found to restore the RNA expression of MIF gene comparable to the control but this trend was not consistent in expression of TNF-α, however, group DM + CMP-1D demonstrated the expression similar to the control (Fig. 6).

## Discussion

Wound healing generally graduates into three phases: an initial inflammatory phase followed by a proliferation phase and finally extracellular matrix formation. Defects in the inflammatory phase under the healing process can cause a failure in the subsequent processes of fibroblast growth and collagen synthesis [25, 26]. The inflammatory phase recruits leukocytes that produce growth factors and remove debris from the wound [27–30]. Our data confirms that CMP has a promising role in the inflammatory phase of wound healing in diabetic models. CMP was found to return wound healing in diabetic rats to a similar level to that of normal rats.

The present investigation vividly demonstrates that diabetic rats have a highly altered redox status, as evidenced by the significantly compromised activity of key antioxidant enzymes/proteins (SOD, CAT and GSH) concomitant with an elevated level of MDA. This clearly suggests that oxidative stress plays a key role in the pathogenesis of diabetes. The severity of this oxidative stress is further exacerbatedin diabetic induced rats even seven days after wounding, which suggests that this stress is also involved in the progression and advancement of diabetes, and potentially increases the suffering experienced by patients. Intriguingly, the proposed peptide has shown a very strong antioxidant potential. The peptide was found to restore the normal redox status in the single dosed treated rats and the situation was also observed to continue to improve in the rats treated with the peptide for a week, such that many of the oxidative parameters were by then near to the control values. It proves that the peptide has a quite significant antioxidant potential although this claim requires further investigation.

IL-1β, TNF-αmodulate the expression of the chemokines and adhesion molecules necessary for the recruitment of inflammatory cells to the site of injury [31]. ELISA analysis of serum TNF-α protein levels showed that it was elevated by diabetes. However, RT-PCR analysis of locally wounded skin tissues revealed that diabetes down-regulated the RNA expression of TNF-α, while CMP was found to significantly restore its RNA expression. The increased TNF-α level in the serum of diabetic wounds might be due to the systemic effect of its pro-inflammatory activities in diabetes [32], while its locally decreased RNA expression in diabetic wounds might logically be responsible for impaired recruitment of inflammatory cells. Similar results were found by Grieb et al., [33] for MIF. Histological examination here confirmed this explanation since the number of inflammatory cells and granulation cells was less in diabetic wounds in comparison to those of the control. Although, neutrophil infiltration peaks within the first six hours in normal wounds [34] diabetes leads to a quantitative reduction in the level of neutrophil infiltration into the wound site up to 24 h after wounding [13]. Additionally, delayed collagen production and only a small number of newly formed blood vessels and disturbed dermis were observed in diabetic wounds. Although it was elevated one day after incision, the NF-kB protein level was significantly decreased in DM + CMP-7D seven days after incision, in comparison to the diabetic one. NF-kB is required for the induction of pro-inflammatory cytokines, such as IL-1β, TNF-α and IL-6 [9]. The oxidative stability induced by CMP may, therefore, mediate the activation of NF-kB, leading to the activation of the inflammatory cascade and the stimulation of wound healing, resulting in faster wound closure rate in CMP rats.

In addition, we have found that diabetes down-regulated the RNA expression of MIF genes both one and seven days after incision in comparison to the control samples under the present investigation. Grieb et al. [32] found that locally decreased levels of MIF in chronic wound exudates might be responsible for impaired recruitment of endothelial progenitor cells. Accordingly, diabetic wounds in this study were delayed in their closure rate in comparison to control wounds. This may be due to the fact that MIF is a critical molecule in pro-inflammatory innate immune responses, being involved in arresting infections [35–38]. From another point of view, these two factors can be considered as a diagnostic biomarker for autoimmune and inflammatory diseases including diabetes [39–41]. Therefore, prolonged elevation of MIF and inflammatory cytokines has been found to be responsible for impaired healing [14].

## Conclusion

CMP has a strong antioxidant potential that reduces the effects of oxygen free radicals and lipid peroxidation by orchestrating the overall antioxidant system to the optimum in vivo. Moreover, CMP is a potential stimulant in normalizing the inflammatory cytokines and restoring high levels of TNF-α mediated by NF-kB in diabetic rats. An increase in neutrophil infiltration at wound sites post CMP administration in diabetic rats speeded up the normal inflammatory events of the healing process. However, investigation of the deep mechanisms in wound healing phases, as well as structural analysis, are necessary to identify the mode of action of this peptide.

## Abbreviations

ALP: Alkaline phosphatase
CAT: Catalase
GSH: Glutathione
MDA: Malondialdehyde
MTS: Mallory Trichrome stain
SOD: Superoxide dismutase
NF-kB: Transcription factor kappa-B
TNF-α: Tumor necrosis factor alpha
IL-1β: Interleukin 1β
