# Erratum to: Camel milk peptide improves wound healing in diabetic rats by orchestrating the redox status and immune response

**DOI:** 10.1186/s12944-017-0421-x

**Published:** 2017-02-20

**Authors:** Hossam Ebaid, Bahaa Abdel-salam, Iftekhar Hassan, Jameel Al-Tamimi, Ali Metwalli, Ibrahim Alhazza

**Affiliations:** 1 Department of Zoology, College of Science, King Saud University, P.O. Box 2455, Riyadh, 11451 Saudi Arabia; 2 Department of Zoology, Faculty of Science, El-Minia University, El-Minia, Egypt; 3 Department of Biology, College of Science and Humanities in Quwiaya, Riyadh, 11961 Saudi Arabia; 4 Department of Food Science, College of Agriculture and Food Science, King Saud University, Riyadh, Saudi Arabia; 5 Department of Dairy, Faculty of Agriculture, El-Minia University, El-Minia, Egypt

## Erratum

After publication of the original article [1], it came to the authors’ attention that an institution had been inadvertently omitted from the Acknowledgements section. The results of the paper are part of a funded project from the National Plan for Sciences, Technology and Innovation (MAARIFAH), King Abdualaziz City for Science and Technology, which should have been acknowledged in the original article.
